# The effect of short-term vitamin D on the antioxidant capacity following exhaustive aerobic exercise

**DOI:** 10.4314/ahs.v23i1.61

**Published:** 2023-03

**Authors:** Vahid Parvizi Mastali, Rastegar Hoseini, Mohammad Azizi

**Affiliations:** Department of Exercise Physiology, Faculty of Sport Sciences, Razi University, Kermanshah, Iran

**Keywords:** Exhaustive Aerobic Exercise, Overweight, Vitamin D, Antioxidant Capacity

## Abstract

**Background:**

Exhaustive Aerobic Exercise (EAE) increases the production of free radicals and oxidative stress in the body. One of the most effective approaches to reduce EAE-induced oxidative stress is antioxidant supplementation.

**Objectives:**

Therefore, the present study investigated the effect of short-term Vitamin D (Vit D) supplementation on antioxidant capacity in inactive men following an EAE session.

**Methods:**

In this clinical trial, 24 non-athlete men were randomly divided into Experimental (Exp; n = 12) and Control (C; n = 12) groups. Exp received 2,000 IU of Vit D daily for six weeks (42 days), while C received a lactose placebo daily with the same color, shape, and warmth percentage. EAE sessions were performed on a treadmill before and after six weeks of supplementation.

**Results:**

The results showed that EAE increased antioxidant capacity and Vit D (P<0.05). Compared with C, six weeks of Vit D supplementation significantly increased superoxide dismutase (P=0.001), glutathione peroxidase (P=0.011), catalase (P=0.018), peroxidase (P=0.001), and Vit D (P=0.001), in the Exp at the Post 2 stage.

**Conclusion:**

Finally, short-term Vit D supplementation could be recommended to non-athlete men deciding to participate in EAE to prevent oxidative damage.

## Introduction

Regular exercise training has been shown to elicit favourable improvements in human health and is known to help prevent and control several diseases such as cardiovascular disease, respiratory disease, Hemodynamic disorders, hypertension, and obesity^1^,^2^. Sedentary lifestyle and obesity are associated with elevated Reactive Oxygen Species (ROS) production that could lead to the chronic state of physiological stress and likely the development of cardiometabolic disease if left unchecked^3^. Also, acute exercise causes a transient increase in ROS production and oxidative stress, yet is believed to elicit favourable improvements^4^. Exercise-induced ROS are believed to augment the response of systemic oxidative stress biomarkers to contraction and exercise by acting as second messengers for skeletal muscle cell signalling 5. However, exercise-induced ROS inhibits proteins, enzymes, and neurotransmitters, induces muscle damage, fatigue, decreases athletic performance, and impairs muscle function^6^ possibly via modifying lipids, proteins, RNA, and DNA, leading to oxidative stress and global cellular damage^7^,^8^. Oxidative stress following exhaustive exercise training leads to alterations in oxidation-reduction (Redox) homeostasis and molecular damage^9^. Despite the destructive effects of oxidative stress, increased ROS elicited by exhaustive exercise is reported to be a requirement for adaptation to physiological stress and optimal cellular functioning^10^. Several studies suggest that exercise might affect antioxidant defence and oxidative stress biomarkers in a FITT-dependent manner (i.e., frequency, intensity, type, and time) which also involve the antioxidant capacity of the individual^11^,^12^. While strenuous aerobic and anaerobic exercise elicits a state of elevated ROS, moderate-intensity regular exercise is beneficial for oxidative stress and health^13^. Thus, organisms have evolved to encompass a complex and interconnected system to adapt and maintain homeostasis.

The underlying mechanisms that increase the effectiveness of the antioxidant system following exercise include adaptation in cellular signalling pathways that stimulate mitochondrial biogenesis and increase muscle oxidative stress capacity 10. Nevertheless, signalling pathways related to skeletal muscle adaptation, specifically with that of mitochondrial biogenesis and endogenous antioxidant upregulation, increase the effectiveness of antioxidant system and the muscle oxidative stress capacity 10. Although the effectiveness has not yet been conclusively determined, athletes have been taking antioxidant supplements to reduce the destructive effects of oxidative stress. Vitamin D (Vit D) supplement involves in the regulation of antioxidant enzymes and deploys antioxidant property via stimulating the expression of several antioxidant defence system molecules including Superoxide Dismutase (SOD), Glutathione Peroxidase (GPX), Catalase (CAT), Peroxidase (POD), and suppressing the NADPH oxidase expression 14, 15. Various studies have also shown that Vit D enhances the strength of the antioxidant defense system by increasing antioxidant capacity and controlling ROS. Vit D reduces ROS and proinflammatory cytokines possibly by improving cellular Glutathione (GSH) levels^16^. In humans however, some studies reported Vit D supplementation to have no or little effect on exercise-induced oxidative stress.

Since there is a discrepancy in the findings, this study aimed to investigate the short-term effect of Vit D supplementation on the antioxidant capacity of non-athlete men following Exhaustive Aerobic Exercise (EAE) sessions.

## Method

### Study design and participants

The trial (IR.RAZI.REC.1400.001) was approved by the Ethics Committee of the Kermanshah Razi University and registered in the Iranian Clinical Trial Registration Center under the code IRCT20210414050965N1; written informed consent was obtained from all participants, including agreement of the patients to participate as volunteers and feasibility to leave the study.

**Figure 1:**
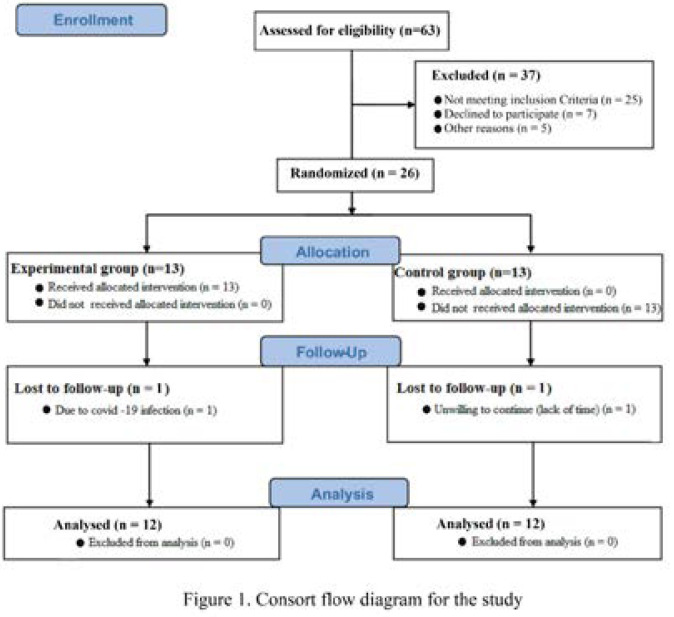
Consort flow diagram for the study

This study is quasi-experimental with pre-test and posttest design and placebo groups. The statistical population of this study was non-athlete men aged 20 to 30 years in Kermanshah. To select the subjects, invitations for participating in the clinical trials were posted on Telegram, Instagram, and WhatsApp. Forty-eight individuals volunteered, among which 26 individuals who met the inclusion criteria were selected as subjects. Inclusion criteria include; Vit D deficiency (25 levels of hydroxyvitamin D below 50 nmol/L), lack of underlying disease, musculoskeletal injury, regular physical activity in the last 6 months, infection with COVID-19, any kind of supplementation, and smoking. One day before the start of the study, the subjects were given the necessary explanations about the research conduction and signed the informed consent of participation in the research. Also, the three-day estimated food record, nutrition (including dietary history, food frequency, eating habits, and behaviours), and health questionnaires were filled. Health questionnaires include; history of previous diseases (hypertension, asthma, vascular problem), family history of diseases, medical history, assessment of physical activity level (activity level, duration of activity), and anthropometric characteristics (weight, height, waist). Subjects were then randomly divided into two groups: Experimental (Exp; n = 13) and Control (C; n = 13); the lottery was used to assign the subjects in the groups. It should be noted that one individual in each group refused to continue the study protocol. Figure 1 shows the consort flow diagram for the study.

### Exhaustive Exercise Protocol

**Figure 2:**
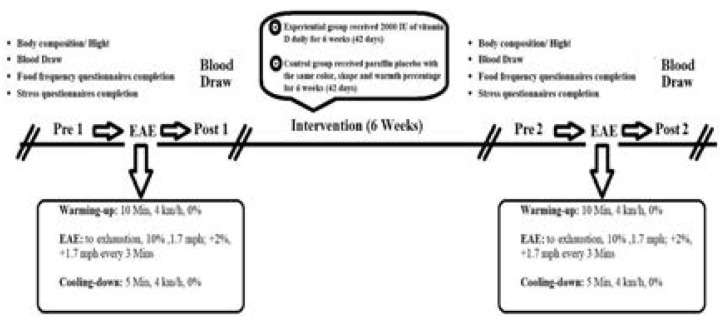
Overview of the study design

Based on previous studies, the exhaustive Bruce aerobic test was executed on a treadmill. After 10 minutes of general warm-up, the subjects started the protocols on the treadmill (10 Min, 4 km/h, 0%). The test consisted of 7 steps of 3 minutes, starting with a 10% slope and 1.7 mph speed. The slope of the device increased by 2%, and the speed increased by 1.7 mph every 3 minutes until the subjects were no longer able to continue their activities and expressed complete exhaustion. Finally, 5 minutes of cooling down at a 4 km/h and a zero slope were performed. While running, the Borg scale Rated Perceived Exertion (RPE) was used (3). Both Exp and C performed the exercise program twice, before starting the Vit D supplementation (or placebo) and after six weeks of intervention (Figure 2).

### Vit D supplementation

The Vit D supplementation group received 2000 IU Vit D per day (Zahravi Pharmaceutical Company) for six weeks (42 days). The C group also received a placebo (containing paraffin made by Zahravi Pharmaceutical Company - Iran), which was similar to Vit D supplements in shape, color, smell, and taste 16 (Figure 2). The subjects completed the 3-days food frequency questionnaire in both pre and post-tests. Subjects were asked to consume the same food and the same number of calories one day before the blood sampling. The subject had a regular diet of 55% carbohydrates, 30% fat, and 15% protein.

Insert Figure 2

### Blood Sampling

The blood samples were taken from the patient four times; the first and second samples were taken at the beginning of the study, before (pre1) and immediately after (post1) the first exercise protocol. The third and fourth samples were taken after six weeks of intervention, before (pre2) and immediately after (post2) the second exercise protocol. For each sampling, 5 ml of blood was taken from the subjects and poured into test tubes and injectors without EDITA. The samples were sent to the laboratory in the frozen state and centrifuged there for 10 minutes at 3000 rpm, and after separating, the serum was stored at -80°C. Finally, the activity of SOD (spectrophotometer, manufactured by Pharmacia Biotech Model 3000, wavelength 560 nm; Merck Germany and Sigma USA kit), GPX (colorimetric method, 412 nm wavelength, Zelbio, Germany kit), CAT (Spectrophotometer, manufactured by Pharmacia Biotech Model 3000; Sigma USA kit), and POD enzymes (spectrophotometer, manufactured by Pharmacia Biotech Model 3000, wavelength 510 nm; Sigma USA kit) were measured.

### Statistical analysis

Descriptive statistics were used to determine the mean and standard deviation of variables, and Shapiro-Wilk test was used to examine the normality of the distribution. Within-group changes were examined using a dependent t-test, while between-group comparisons were made using a repeated-measures ANOVA, Bonferroni's post hoc test. All analyses were performed with SPSS software (version 26) at a P<0.05.

## Results

The anthropometric characteristics of the subjects are presented in table 1. It shows that Bodyweight (BW), Body Mass Index (BMI), Percent Body Fat (PBF), and Waist Hip Ratio (WHR) were significantly reduced in Exp. However, these variables increased in the C; however, not statistically significant (except in PFB; P=0.041). Also, the results of the independent t-test showed significant differences in BW (P=0.039), BMI (P=0.025), PBF (P=0.043), and WHR (P=0.035), comparing the pre-test to post-test alterations between Exp and C.

**Figure 3:**
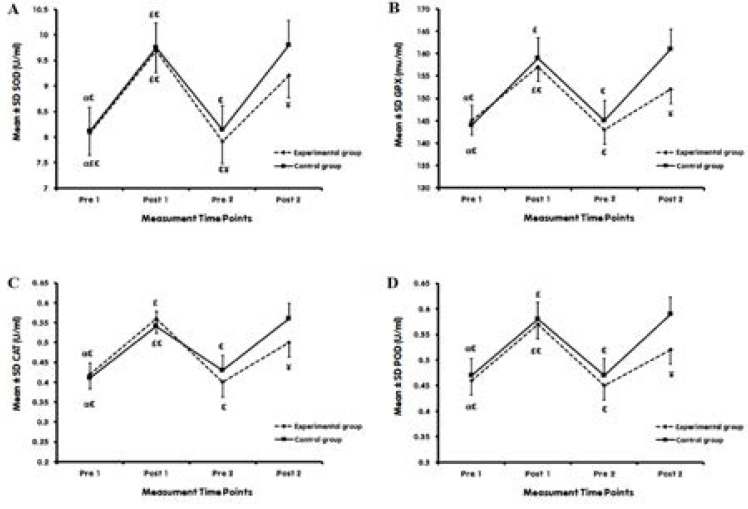
Antioxidant capacity levels at different time points in Exp and C **BW:** Bodyweight; **BMI:** Body Mass Index; **PFB:** Percent Body Fat; **WHR:** Waist-Hip Ratio P values superscript with “a” is calculated using an independent t-test for comparing Δ between groups. P values superscript with “b” is calculated using dependent t-test for comparing pre-test and post-test within groups

The present study results indicate a significant increase in antioxidant capacity (SOD, GPX, CAT, and POD) in both C and Exp following the EAE (comparing Pre 1 and Post 1). Also, after six weeks of Vit D supplementation, the antioxidant capacity increased significantly in both C and Exp following EAE (comparison of Pre 2 and Post 2). However, in Exp, the increase in SOD in Post 2 was significantly higher than in Post 1; Also, GPX, CAT, and POD were increased in Post 2 compared to Post 1, but not statistically significant (Figure 3).

The independent t-test showed significantly higher SOD, GPX, CAT, and POD in the Exp compared with C in Post 2. While no such differences were observed between Exp and C in variables mentioned above in Pre 1 and Post 1, and Pre 2 (except SOD; P=0.033) (Figure 3).

**Figure 4:**
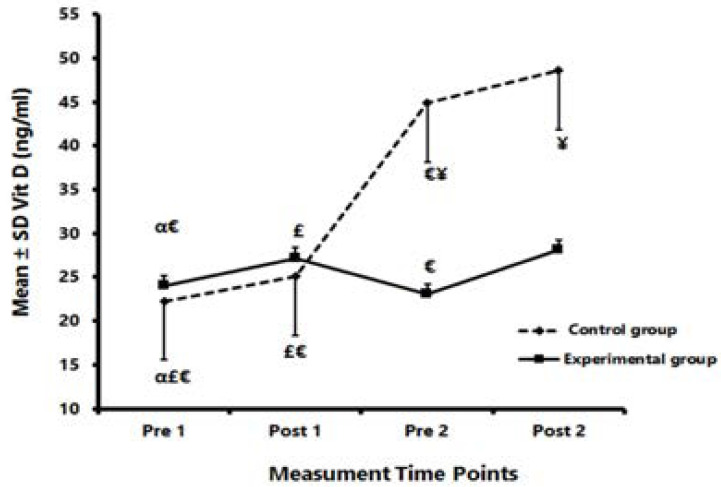
Vit D levels at different time points in Exp and C α: Significantly different compared with Post 1. £: Significantly different compared with Pre 2. €: Significantly different compared with Post 2. ¥: Significantly different comparing Exp and C.

Figure 4 shows a significant increase in Vit D in both C and Exp following the EAE (comparing Pre 1 and Post 1). After six weeks, Vit D levels increased significantly in Exp, while a significant decrease was observed in C (comparison of Pre 1 and Pre 2).

Also, Vit D increased significantly in both C and Exp following the second EAE session (comparison of Pre 2 and Post 2). However, in Exp, the increase in Vit D in Post 2 was significantly higher than in Post 1. The independent t-test showed significantly higher Vit D in the Exp compared with C in Pre 2 (P=0.001) and Post 2 (P=0.001) (Figure 4).

## Discussion

This study aimed to evaluate the effect of short-term Vit D supplementation on the EAE-induced antioxidant capacity alteration in non-athlete young men. Consistent with the present study, Taherkhani et al. (2019) reported a significant increase in antioxidant capacity, including SOD, GPX, CAT, and POD enzymes, after a single EAE session 17. Also, Nobari et al. (2021) reported that high-intensity exercise causes significantly increased oxidative stress and thus increases total antioxidant capacity 18. On the one hand, EAE modifies the metabolism of prostanoids, Xanthine Oxidase, NADPH oxidase, and macrophage activity by increasing catecholamines secretion 19. On the other hand, EAE leads to impaired calcium ion homeostasis, damaged iron-containing proteins, and the production of xanthine oxidase, which in turn produces ROS and increases oxidative stress^20^,^21^. The results of the present study showed that short-term Vit D supplementation increases antioxidant capacity and reduces oxidative stress significantly following EAE. In contestant to the present study, Ke et al. (2016) reported that Vit D reduces oxidative stress following strenuous exercise 22. Also, Jafari et al. (2021) reported that Vit D supplementation and exercise modulate antioxidant defense and the expression of antioxidant genes 23. Vit D is also considered an antioxidant that might cause the expression of several genes in the antioxidant defense system, including GPX, GSH, CAT, SOD, and the suppression of NADPH oxidase 24, 25. Besides, increasing Vit D levels in individuals with insufficient Vit D levels (trained and untrained) have improved antioxidant capacity 26. All in all, Vit D deficiency negatively affects antioxidant capacity, and Vit D supplementation upregulates the activity of SOD and CAT 27.

## Conclusion

Generally, short-term Vit D supplementation downregulates the oxidative stress following EAE. Therefore, Vit D supplementation could be recommended to non-athletes to increase their antioxidant capacity.

## Limitation of the study

Thus, increased antioxidant capacity following six weeks of Vit D supplementation might be due to the antioxidant role of Vit D. The study can be carried out using a larger sample size. The long-term effect of Vit D is recommended to be investigated.

## Data Availability

All data generated or analysed during this study are available from the corresponding author on reasonable request.

## Abbreviations

EAE: Exhaustive Aerobic Exercise
Vit D: Vitamin D
SOD: Superoxide dismutase
GPX: Glutathione peroxidase
CAT: Catalase
POD: Peroxidase
BW: Body Weight
BMI: Body Mass Index
PFB: Percent Body Fat
WHR: Waist-Hip Ratio
